# Comparative morphological and lipidomic profiling of abdominal adipose tissue in Beijing You chickens at different ages and Ross 308 broilers

**DOI:** 10.1016/j.psj.2025.105613

**Published:** 2025-07-26

**Authors:** Yaxi Xu, Keyi Xu, Luyao Wang, Liyang Zhu, Cheng Long, Zili Lin, Jinhuan Dou, Xiangguo Wang, Xiaolong Qi, Li Chen, Xihui Sheng

**Affiliations:** a College of Animal Science and Technology, Beijing University of Agriculture, Beijing 102206, PR China; b College of Food Science and Engineering, Beijing University of Agriculture, Beijing 102206, PR China

**Keywords:** Abdominal fat, Lipidomic, Beijing You chicken, Ross 308 broiler

## Abstract

This study aimed to compare fat deposition, muscle tissue structure, and abdominal fat lipid metabolism among 150-day-old Beijing You chickens, 450-day-old Beijing You chickens, and 150-day-old Ross 308 broilers. Slaughter performance analysis revealed that Beijing You chickens exhibited significantly higher fat deposition than Ross 308 broilers, with continuous accumulation observed with increasing age. Histological observations showed that the breast muscle fibers of Beijing You chickens were significantly smaller than those of Ross 308 broilers of the same age, while the diameters of abdominal adipocytes were significantly larger and increased further with age. Lipidomic profiling of abdominal fat identified 613 significantly different lipid species between Beijing You chickens and Ross 308 broilers, and 250 lipid species differing between the two age groups of Beijing You chickens. Among them, 123 lipid species were differentially abundant in both comparisons, mainly enriched in glycerophospholipid and sphingolipid metabolism pathways, suggesting their key roles in lipid metabolic remodeling. These findings reveal distinct differences in lipid metabolism and muscle development among chicken breeds and provide a theoretical basis for understanding the molecular mechanisms underlying flavor, tenderness, and metabolic regulation, as well as a valuable reference for molecular breeding of high-quality poultry.

## Introduction

The poultry industry represents a critical component of global agriculture, serving as a primary source of high-quality protein and making substantial contributions to global food security and nutritional health (Ge et al., 2023). With the continuous expansion of the global population and the ongoing improvement of living standards, consumer demand for meat products, particularly high-quality and flavorful poultry, has been escalating. This trend necessitates that the development of the poultry industry not only focuses on enhancing production efficiency but also addresses critical challenges related to sustainability and animal welfare (Chen et al., 2023).

Among various poultry breeds, Beijing You chicken and Ross 308 broiler have garnered significant attention due to their distinct characteristics. Beijing You chicken, renowned for its superior meat quality and traditional rearing methods, has gained substantial popularity in the Chinese market (Gao et al., 2025; Ge et al., 2023). In contrast, Ross 308 broiler has established itself as a cornerstone of the global commercial poultry industry, primarily due to its exceptional growth rate and high feed conversion efficiency (Biesiada-Drzazga et al., 2017; Eila et al., 2012). The contrasting features of these two breeds not only cater to the diverse demands of the market but also provide valuable opportunities for comparative research in areas such as meat quality, fat deposition, and lipid composition.

Meat quality, a critical determinant of consumer preference for poultry products, is influenced by a multitude of factors, including genetic predisposition, dietary composition, age at slaughter, and adipose tissue content (Baéza et al., 2022; Muktar et al., 2021). Adipose tissue, particularly abdominal fat, plays a pivotal role in determining both the nutritional value and sensory attributes of poultry meat (Luo et al., 2022; Ma et al., 2021; Xiang et al., 2021). The variations in fat deposition patterns and lipid composition among different poultry breeds not only impact meat quality characteristics but also reflect underlying differences in genetic makeup and environmental influences (Cao et al., 2023). While, age is one important factors associated with meat flavors in chicken, especially in many native breeds, such as Beijing You chicken (Ge et al., 2023; Li et al., 2019; Yuan et al., 2022). Many consumers, especially in Chinese market, prefer older chicken because they believe that older chicken have better flavor and nutrition (Xiao et al., 2019). Previous study have found that the age of chicken could affect both fatty composition and related metabolism in breast meat (Zhang et al., 2022). The 150 and 450 days represent the early and late laying stages, respectively, of Beijing-You chickens (Zhang et al., 2023). These two age groups are the predominant market forms of the breed and cater to distinct consumer preferences due to differences in flavor profile.

With the continuous advancement of modern animal husbandry, the rising consumer demand for premium-quality poultry meat has spurred researchers to undertake comprehensive investigations into the genetic characteristics and growth dynamics of poultry species. These studies aim to optimize breeding strategies and refine feed formulations (Dong et al., 2024; Kleyn and Ciacciariello, 2021; Saleque and Absarey, 2020). This study focuses on comparisons of carcass traits, meat quality, histological morphology, and lipidomic profiles of abdominal adipose tissue in Beijing You chickens at 150 and 450 days of age and Ross 308 broilers at 150 days of age. The objective is to identify key lipid species and metabolic pathways linked to fat deposition and meat quality differences between the two breeds at different developmental stages. By integrating analyses of carcass performance, meat quality, tissue morphology, and lipids composition, the study aims to explain age-related and breed-specific variations in fat deposition and their effects on meat quality. The findings will inform breeding programs to optimize fat deposition and enhance meat quality, supporting sustainable poultry industry development.

## Materials and methods

### Ethics Statement

#### Animals and samples collection

All experimental procedures and animal welfare practices were conducted in accordance with the Guide for the Care and Use of Laboratory Animals. All procedures were reviewed and approved by the Animal Ethics Committee of the Beijing University of Agriculture (Approval ID: BUA-zc-20200073).

To standardize physiological states prior to sample collection, ten female 150-day-old Beijing You chickens, ten female 450-day-old Beijing You chickens, and ten female 150-day-old Ross 308 broilers were raised under identical conditions for one week. During this period, all birds had free access to feed and water, were maintained under natural light, and housed at a room temperature of 28–32 °C. The basal diet was formulated according to the nutritional requirements for Beijing You chickens, with a metabolizable energy content of approximately 12.0 MJ/kg and crude protein content of 16.5 %. The diet also contained adequate levels of essential amino acids, vitamins, and minerals to support maintenance and growth. Following a 12-hour feed withdrawal, each bird was individually weighed, and blood samples were collected from the wing vein. The chickens were then euthanized, and tissue samples of abdominal fat, liver, breast muscle, and leg muscle were promptly collected.

The abdominal fat tissue was divided into two portions: one was immediately frozen in liquid nitrogen and stored at −80°C for subsequent lipidomic analysis, while the other was sectioned into 1 × 1 × 1 cm cubes, washed with physiological saline to remove residual blood and then fixed in 4 % paraformaldehyde for morphological examination. Similarly, breast muscle and leg muscle tissues were each divided into two portions: one was stored at 4°C for short-term preservation, and the other was sectioned into 1 × 1 × 1 cm cubes, rinsed with physiological saline, and fixed in 4 % paraformaldehyde for morphological examination.

### Traits measurement

Meat production traits were measured before or immediately after slaughter. The evaluated traits encompassed body weight, carcass weight, liver weight, breast meat weight, leg meat weight, skin weight, and abdominal fat weight. Furthermore, the ratios of breast meat weight, leg meat weight, skin weight and abdominal fat weight to carcass weight were calculated as the breast meat rate, leg meat rate, skin rate and abdominal fat rate, respectively.

The meat quality attributes, including pH levels, meat coloration, and shear force of both breast and thigh muscles, were rigorously evaluated post-slaughter. The pH of the breast and thigh muscles was measured approximately 45 minutes after slaughter using a high-precision pH meter with an accuracy of ±0.01 units, equipped with a durable pH probe. The probe was inserted into the left muscle tissue of each specimen at three predetermined locations within each muscle, and pH readings were taken. The average of these triplicate measurements was calculated and recorded as the final pH value.

Meat coloration for both the breast and thigh muscles was quantified using a sophisticated colorimeter, which measured the L* value (indicative of lightness), a* value (denoting redness), and b* value (representing yellowness). These measurements were conducted at three distinct sites on the left muscle of each specimen, and the mean of these triplicate assessments was recorded as the final color metric.

The shear force of the breast and thigh muscles was evaluated according to the established agricultural industry standard (NY/T1180-2006). From the left side of each specimen, samples of both the breast and thigh muscles were excised. A cylindrical sampler with a diameter of 1.27 cm was used to extract meat columns aligned with the muscle fibers orientation. Three columns were obtained from each muscle sample, and each column underwent a single shear force measurement. The mean of these three measurements was calculated and recorded as the final shear force value.

### Histological examination of fat and muscle tissues

The 1 cm^3^ blocks of adipose and muscle tissue samples collected from fresh tissue were fixed in 4 % paraformaldehyde for more than 24 hours (adipose tissue requires longer fixation due to its high lipid content). After fixation, the tissues were dehydrated through a graded ethanol series (75 %, 85 %, 95 %, and 100 %), then cleared in xylene to replace water and lipids, facilitating paraffin infiltration. The tissues were then immersed in molten paraffin and embedded in embedding molds, followed by cooling for 2 hours to form paraffin blocks. Sections of 5 micrometers in thickness were cut using a microtome, floated on warm water to flatten, mounted onto glass slides, and baked at 60℃ for 40 minutes to ensure firm adhesion.

Prior to hematoxylin and eosin (H&E) staining, the sections were deparaffinized in xylene and rehydrated through a descending alcohol series to distilled water. The sections were stained with hematoxylin for 10 minutes to visualize cell nuclei in bluish-purple, followed by differentiation with acid alcohol for 3 seconds to remove non-specific staining. A bluing step with alkaline solution was then performed to enhance nuclear clarity. Subsequently, eosin was applied for 3 minutes to stain the cytoplasm and other structures pink. After staining, the sections were dehydrated through graded alcohols, cleared in xylene, and mounted with neutral resin.

The stained sections were examined and photographed under an optical microscope. Cell diameters were measured using Image Pro Plus software, with more than 1,000 cells measured per individual to calculate the average cell diameter for that sample.

### Lipidomic sequencing

Lipids were extracted from abdominal adipose tissues using the methyl tert‑butyl ether (MTBE) method (Matyash et al., 2008). Specifically, 20 mg of adipose tissue samples were thawed on ice and homogenized in 1 mL of methanol/MTBE (1:3, v/v) containing an internal standard for 15 minutes at 210 rpm on a roller mixer. Subsequently, 200 μL of ddH_2_O was added to the mixture, followed by incubation for 1 minute at 210 rpm on the roller mixer. The mixture was then centrifuged at 12,000 rpm and 4°C for 10 minutes to facilitate phase separation. A 300 μL aliquot of the upper organic phase was collected, dried under nitrogen gas, and reconstituted in 200 μL of mobile phase B. The reconstituted solution was stored at −80°C until further analysis. Finally, the prepared samples were transferred to sample bottles for subsequent LC-MS/MS analysis.

Lipid profiling, identification, and quantification were performed as described below, following the protocol from a previous study (Breitkopf et al., 2017). Specifically, lipid profiling was conducted using an LC-ESI-MS/MS system that integrates ultra-performance liquid chromatography (UPLC) with tandem mass spectrometry (MS/MS). The UPLC system used was the SCIEX ExionLC AD (Danaher Corporation, USA), while the MS/MS system was the SCIEX QTRAP ® LC-MS/ MS (Danaher Corporation, USA).

The UPLC analytical conditions were as follows: A Thermo Accucore™ C30 column (2.6 μm particle size, 100 mm × 2.1 mm inner diameter) was employed. The mobile phase composition was as follows:

Mobile phase A: acetonitrile/water (60/40, V/V) containing 0.1 % formic acid and 10 mmol/L ammonium formate; Mobile phase B: acetonitrile/isopropanol (10/90, V/V) containing 0.1 % formic acid and 10 mmol/L ammonium formate.

The gradient elution program was set as follows: 0 min, 80 % A (20 % B); 2 min, 70 % A; 4 min, 40 % A; 9 min, 15 % A; 14 min, 10 % A; 15.5 min, 5 % A; 17.3 min, 5 % A; Returned to initial conditions (80 % A) at 17.3 min and maintained until 20 min.

The flow rate was 0.35 mL/min, the column temperature was maintained at 45°C, and the injection volume was 2 μL. The effluent was directed to an ESI-triple quadrupole-linear ion trap (QTRAP)-MS for detection.

Combined linear ion trap (LIT) and triple-quadrupole (QqQ) scans were conducted on a QTRAP® LC-MS/MS system equipped with an ESI Turbo Ion-Spray interface. The system operated in both positive and negative ion modes and was controlled by Analyst 1.6.3 software (Sciex, Framingham, MA). The ESI source parameters were configured as follows:

Ion source type: turbo spray; Source temperature: 500 ℃; Ion spray voltage (IS): 5500 V in positive mode and −4500 V in negative mode; Ion source gas 1 (GS1): 45 psi; Gas 2 (GS2): 55 psi; Curtain gas (CUR): 35 psi; Collision gas (CAD) pressure: medium.

Instrument tuning and mass calibration were conducted using 10 μmol/L and 100 μmol/L polypropylene glycol solutions in QqQ and LIT modes, respectively. QqQ scans were acquired as multiple reaction monitoring (MRM) experiments with the collision gas (nitrogen) set to 5 psi. The declustering potential (DP) and the collision energy (CE) for individual MRM transitions were optimized through a systematic process. Specific sets of MRM transitions were monitored during each time period based on the metabolites eluted within that interval.

### Lipidomic analyses

Unsupervised principal component analysis (PCA) was conducted using the prcomp function in R version 4.1.2(Holland, 2008). Before the analysis, the data were scaled to unit variance.

Hierarchical cluster analysis (HCA) results of samples and metabolites were visualized as heatmaps with dendrograms. Pearson correlation coefficients (PCC) between samples were calculated using the cor function in R version 4.1.2 and presented as heatmaps (Becker, 2018). Both HCA and PCC analyses were performed using the R package ComplexHeatmap v2.9.4 (Gu, 2022). For HCA, the normalized signal intensities of metabolites (scaled to unit variance) were visualized as a color spectrum.

For two-group analysis, differential metabolites were identified based on VIP (Variable Importance in Projection) values (VIP > 1) and P-values (*P* < 0.05, Student’s t-test). VIP values were extracted from OPLS-DA result, which included score plots and permutation plots, using the R package MetaboAnalystR v1.0.1 (Chong and Xia, 2018). The data were log-transformed (log2) and mean-centered prior to OPLS-DA. To prevent overfitting, a permutation test with 200 permutations was conducted.

Identified metabolites were annotated using the KEGG Compound database (http://www.kegg.jp/kegg/compound/), and subsequently mapped to the KEGG Pathway database (http://www.kegg.jp/kegg/pathway.html). Pathways with significantly regulated metabolites were subjected to metabolite sets enrichment analysis (MSEA), and their significance was determined using hypergeometric test p-values.

### Statistical analysis

Statistical analyses were conducted on various parameters measured across the three groups. Single-factor analysis of variance (ANOVA) was performed using SPSS software, followed by post-hoc Duncan’s multiple range test for multiple comparisons.

## Results

### Comparisons of slaughter traits between Beijing You chickens of different ages and Ross 308 broilers

The carcass traits of Beijing You chickens of different ages (150 and 450 days) and 150-day-old Ross 308 broilers were statistically analyzed, as presented in Table 1. Significant differences were observed among the three groups.

**Table 1:** Slaughter traits between Beijing You chickens of different ages and Ross 308 broilers.

	Ross 308 broiler at 150 days of age	Beijing You chicken at 150 days of age	Beijing You chicken at 450 days of age
Body weight/g	2130.75 ± 99.58^a^	1491.40 ± 193.88^c^	1911.20 ± 294.93^b^
Carcass weight/g	2016.70 ± 100.10^a^	1365.70 ± 186.96^c^	1776.10 ± 291.34^b^
Abdominal fat weight/g	6.17 ± 5.45^b^	29.50 ± 15.50^b^	97.61 ± 57.88^a^
Abdominal fat rate/%	0.30 ± 0.27^c^	2.09 ± 0.92^b^	5.32 ± 2.52^a^
Skin weight/g	86.20 ± 10.90^b^	118.31 ± 32.43^b^	198.81 ± 71.54^a^
Skin rate/%	4.27 ± 0.41^c^	8.60 ± 1.65^b^	11.04 ± 2.47^a^
Breast meat weight/g	223.20 ± 21.13^a^	89.72 ± 16.93^b^	99.37 ± 19.58^b^
Breast meat rate/%	10.47 ± 0.77^a^	6.00 ± 0.69^b^	5.18 ± 0.56^c^
Leg meat weight/g	346.50 ± 30.54^a^	92.88 ± 19.42^b^	108.79 ± 21.23^b^
Leg meat rate/%	16.26 ± 1.16^a^	6.20 ± 0.79^b^	5.67 ± 0.47^b^
Liver weight/g	40.30 ± 7.19^a^	27.62 ± 6.55^b^	28.57 ± 3.43^b^
Liver_rate/%	2.00 ± 0.34^a^	2.02 ± 0.36^a^	1.63 ± 0.22^b^

Different letters (a, b, c for *p* < 0.05) indicate significant differences.

Ross 308 broilers exhibited significantly higher body weight, carcass weight, breast meat weight, breast meat rate, leg meat weight, leg meat rate, and liver weight compared to Beijing You chickens. In contrast, abdominal fat weight, abdominal fat rate, skin fat weight, and skin fat rate were significantly lower in Ross 308 broilers than in Beijing You chickens. These results highlight the greater fat deposition capacity and slower growth pattern of Beijing You chickens.

In the 450-day-old Beijing You chickens, significant increases were observed in body weight, carcass weight, abdominal fat weight, abdominal fat rate, skin fat weight, and skin fat rate compared to the 150-day-old group. However, no significant changes were noted in traits related to breast and leg muscles or liver weight. These results suggest that, during the later stages of growth, Beijing You chickens exhibit a marked increase in fat deposition, while the growth of breast and leg muscles appears to plateau or increase at a slower rate without reaching statistical significance.

### Comparison of meat quality traits between Beijing You chickens of different ages and Ross 308 broilers

A comparative study was conducted to analyze pH, meat color, and shear force characteristics of the breast and leg muscles of Beijing You chickens at two different ages and 150-day-old Ross 308 broilers. The results are summarized in Table 2.

**Table 2:** Quality traits of breast meat and leg meat of Beijing You chickens of different ages and Ross 308 broilers.

	Parameter	Ross 308 broiler at 150 days of age	Beijing You chicken at 150 days of age	Beijing You chicken at 450 days of age
Breast meat	pH (24 h)	5.94 ± 0.14^b^	11.54 ± 0.78^a^	12.07 ± 1.03^a^
	L* (lightness)	46.87 ± 5.17^a^	42.51 ± 2.30^b^	42.88 ± 3.74^b^
	a* (redness)	0.39 ± 1.03^b^	4.75 ± 2.92^a^	4.21 ± 2.11^a^
	b* (yellowness)	3.91 ± 2.06^b^	7.49 ± 3.11^a^	8.75 ± 2.26^a^
	Shear force (N)	33.86 ± 8.42	26.71 ± 11.65	32.01 ± 9.79
Leg meat	pH (24 h)	6.06 ± 0.11^b^	11.90 ± 1.29^a^	11.52 ± 1.50^a^
	L* (lightness)	50.69 ± 5.33^a^	38.48 ± 3.13^b^	36.49 ± 3.19^b^
	a* (redness)	2.94 ± 2.53^b^	21.39 ± 4.99^a^	22.45 ± 3.86^a^
	b* (yellowness)	0.34 ± 1.15^b^	8.48 ± 3.43^a^	8.63 ± 2.67^a^
	Shear force (N)	60.85 ± 9.46^a^	32.01 ± 9.54^c^	42.88 ± 14.86^b^

L* measures darkness to lightness (greater L* indicates a lighter color), a* measures redness (greater a* indicates a redder color), b* measures yellowness (greater b* indicates a more yellow color); shear force measures maximum peak force (N). Different letters (a, b, c for *p* < 0.05) indicate significant differences.

As seen in Table 1, the pH of breast meat in Beijing You chickens was significantly higher compared to Ross 308 broilers. Additionally, Beijing You chickens demonstrate a significantly lower meat color L value (lightness), along with significantly higher values for both the a value (redness) and b value (yellowness). Moreover, the shear force of the breast meat was notably lower in Beijing You chickens than in Ross 308 broilers. Significant differences were also observed in meat quality traits such as pH, meat color, and shear force within different age groups of Beijing You chicken breast meat. With increasing age, there was a decrease in pH levels and L values, while the a value increased; however, no significant change was observed for the b value. Notably, shear force increased substantially with age. These findings suggest that Beijing You chicken breast meat exhibits superior tenderness compared to Ross 308 broilers; however, older Beijing You chickens display reduced tenderness compared to younger ones, which may be attributed to intramuscular fat deposition.

The leg muscle pH of Beijing You chickens was significantly higher than that of Ross 308 broilers, as indicated in Table 2. Additionally, the meat color L value of Beijing You chickens was significantly lower, while the a and b values were significantly higher compared to Ross 308 broilers. The shear force of the leg muscle was also significantly lower in Beijing You chickens. Significant differences were observed in meat pH, meat color, and shear properties among different age groups of Beijing You chickens. As age increased, there was a decrease in pH and meat color L value, an increase in the a value with no significant change in the b value, and a significant increase in shear force. These results demonstrate that the tenderness of leg muscles is greater in Beijing You chickens compared to Ross 308 broilers and decreases with increasing age. These findings are consistent with those related to pectoral muscle traits.

### Comparison of tissue morphology between Beijing You chickens of different ages and Ross 308 broilers

HE staining was performed to observe the cell morphology of breast meat, leg muscle, and abdominal fat tissue. The cell width of muscle and adipose cells was measured.

**Fig. 1:**
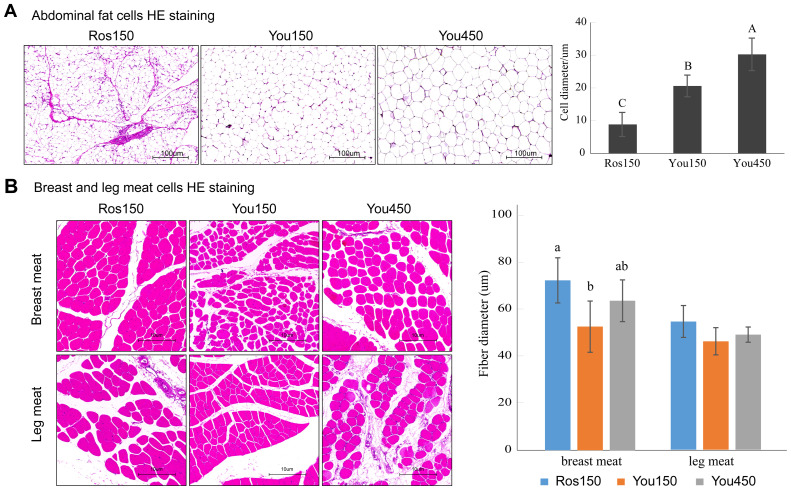
Effects of age and breed on the histomorphology of abdominal fat, liver, and muscle tissues. A. The comparison of abdominal fat cells HE Staining among Ros150, You150, and You450 groups. B. The comparison of breast and leg meat fiber diameter among Ros150, You150, and You450 groups. Ros150 means Ross 308 broilers at 150 days of age; You150 means Beijing You chickens at 150 days of age, and You450 means Beijing You chickens at 450 days of age. Different letters (A, B, C for *p* < 0.01 and a, b, c for *p* < 0.05) indicate significant differences.

The abdominal fat cell diameter was compared among Ross 308 chickens at 150 days of age (Ros150), Beijing You chickens at 150 days of age (You150), and Beijing You chickens at 450 days of age (You450) (Fig. 1A). The abdominal fat cell diameter of the You450 group was significantly (*p* < 0.01) larger than that of the You150 group, and the abdominal fat cell diameter of the You150 group was significantly (*p* < 0.01) larger than that of the Ros150 group. This indicates stronger lipid deposition in abdominal fat tissues of Beijing You chickens compared to Ross 308 broilers, with lipids accumulation increasing with age.

The muscle fiber diameter of leg meat and breast meat was compared among the three groups (Fig. 1B). The fiber diameter of leg meat did not show significant difference among the groups, while the breast muscle fiber diameter of Ross 308 broilers was significantly (*p* < 0.05) larger than that of Beijing You chickens. The breast meat fiber diameter of Beijing You chickens did not show significant differences with increasing age.

### Comparison of lipids composition between Beijing You chickens of different ages and Ross 308 broilers

**Fig. 2:**
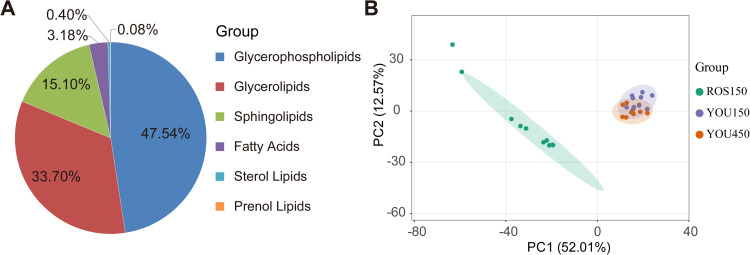
Lipids composition and lipid composition variations among Ros150, You150, and You450 groups. A. lipids composition in abdominal fat tissues of all chickens. B. Principal component analysis (PCA) plots depicting the lipid composition variations among Ros150, You150, and You450 groups. Ros150 represents abdominal adipose tissues obtained from 150-day-old Ross 308 broilers; You150 represents abdominal adipose tissues obtained from 150-day-old Beijing You chickens; You450 represents abdominal adipose tissues obtained from 450-day-old Beijing You chickens.

A total of 1258 lipids were detected in the abdominal adipose tissues of the 30 chickens, including 40 fatty acids, 424 glycerolipids, 598 glycerophospholipids, 1 prenol lipid and 5 sterol lipids (Fig. 2A and Supplementary Table 1-2). Principal component analysis (PCA) was conducted on the measured data of subcutaneous fat tissues from different breeds of chickens, followed by a difference analysis to compare the variability between and within-group samples.

The lipid composition of abdominal fat in Ross 308 broilers and Beijing You chickens exhibits pronounced disparities; however, the variation in lipid composition among different ages of Beijing You chickens is relatively insignificant (Fig. 2B).

**Fig. 3:**
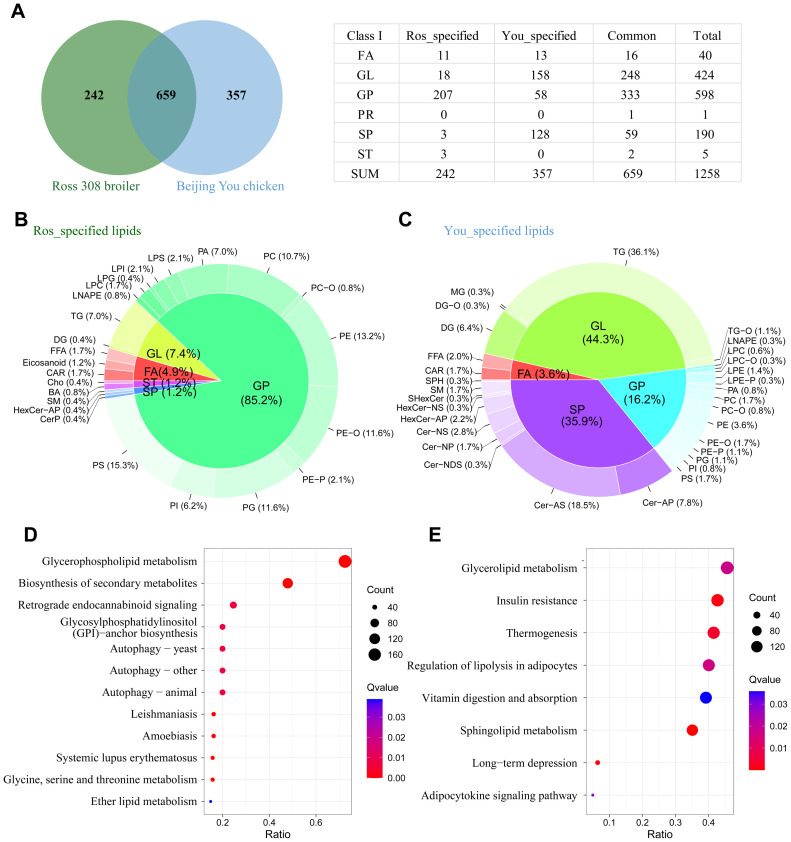
Breed-specific lipids and pathway enrichment in Ross 308 broilers and Beijing You chickens. A. Number of shared and breed-specific lipids and class I lipids, including fatty acids (FA), glycerolipids (GL), glycerophospholipids (GP), phospholipids (PR), sphingolipids (SP), and sterol lipids (ST) in Ross 308 broilers and Beijing You chickens. B. Composition of Ross 308 Broiler specified lipids. C. Composition of Beijing You chicken specified lipids. D. Pathway enrichment of Ross 308 broiler specific lipids. E. Pathway enrichment of Beijing You chicken specific lipids.

Among the 1258 lipids detected, 659 species were commonly presented in the abdominal fat of Beijing You Chickens and Ross 308 broilers, including 16 fatty acids (FA), 248 glycerolipids (GL), 333 glycerophospholipids (GP), 1 prenol lipid (PR), 59 sphingolipids (SP), and 2 sterol lipids (ST). Additionally, 242 lipid species were specific to Ross 308 broilers, including 11 FA, 18 GL, 207 GP, 3 SP, and 3 ST. Furthermore, 357 lipid species were specific to Beijing You chickens, including 13 FA, 158 GL, 58 GP, and 128 SP (Fig. 3A and Supplementary Table 2-5).

The lipid species specific to Ross 308 broilers comprised 85.2 % GP, 7.4 % GL, 4.9 % FA, 1.2 % ST, and 1.2 % SP (Fig. 3B); while those specific to Beijing You chickens were mainly GL (44.3 %), followed by SP (35.9 %), GP (16.2 %), and FA (3.6 %) (Fig. 3C).

Enrichment analysis revealed that the 242 lipid species specific to Ross 308 broilers were primarily enriched in glycerophospholipid metabolism, biosynthesis of secondary metabolites, retrograde endocannabinoid signaling, glycosylphosphatidylinositol (GPl) -anchor biosynthesis, autophagy, leishmaniasis, amoebiasis, systemic lupus erythematosus, glycine, serine and threonine metabolism and ether lipid metabolism pathways (Fig. 3D and Supplementary Table 6). In contrast, the 357 lipid species specific to Beijing You chickens were primarily enriched in glycerolipid metabolism, insulin resistance, thermogenesis, regulation of lipolysis in adipocytes, vitamin digestion and absorption, sphingolipid metabolism, long term depression, and adipocytokine signaling pathways (Fig. 3E and Supplementary Table 7).

Differential Analysis of Abdominal Fat Lipids Between the You450 and You150 Groups

**Fig. 4:**
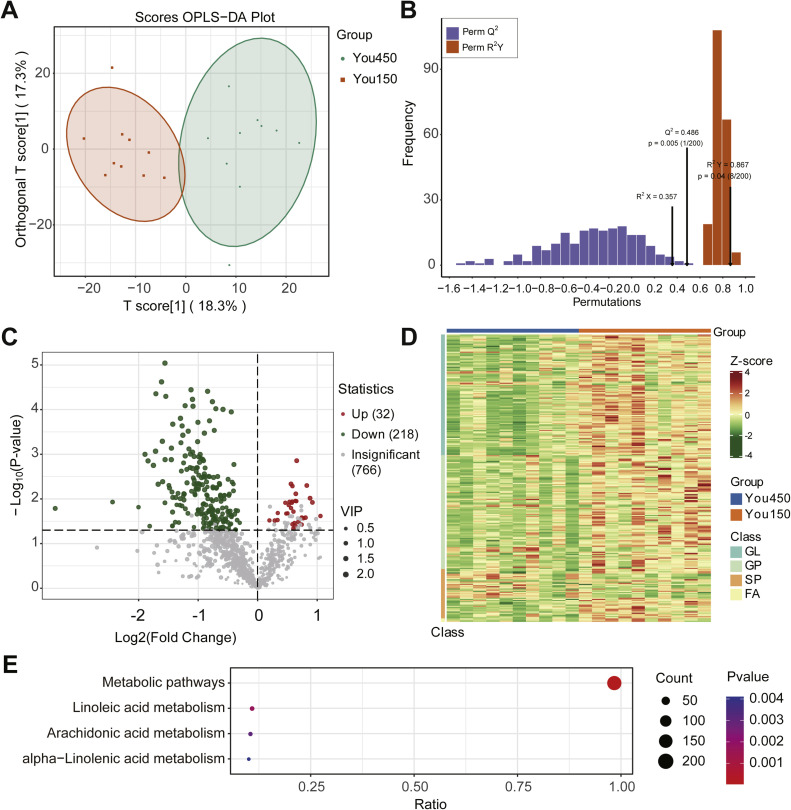
Differential lipids identification between Bejing You chicken at 150 days of age (You150) and 450 days of age (You450). A. Orthogonal partial least squares discriminant analysis (OPLS-DA): OPLS-DA model. B. OPLS-DA: the permutation test. C. Volcano plot of the You450 vs You150. D. Heat map depicting differential lipids in You450 vs You150. E. KEGG enrichment of differential lipids detected between You450 group and You150 group. GL: glycerolipids; GP: glycerophospholipids; SP: sphingolipids; FA: fatty acids.

Orthogonal Partial Least Squares-Discriminant Analysis (OPLS-DA) was conducted to evaluate the differences in lipidomic profiles between the You450 and You150 groups. The score plot (Fig. 4A) showed a clear separation between the two groups, with the first component (T score [1]) explaining 18.3 % of the variance and the orthogonal component (Orthogonal T score [1]) explaining 17.3 %. This indicates a significant distinction in the lipid composition between the two groups.

Model validation using permutation test confirmed the reliability of the OPLS-DA model (Fig. 4B), with $R^{2}X=0.357$, $R^{2}Y=0.867\left(p=0.04\right)$, and $Q^{2}=0.867\left(p=0.005\right)$. A total of 250 lipid species were significantly different, including 32 upregulated lipids and 218 downregulated lipids in You450 group (VIP > 1, *p* < 0.05) (Fig. 4C and Supplementary Table 8). The majority of these differentially expressed lipids were phospholipids (GP) and glycerolipids (GL), suggesting that these lipid classes may contribute substantially to the observed lipidomic differences. Among these 250 differential expressed lipids, only 80 lipids (including 79 downregulated lipids and 1 upregulated lipid in You450 group) differed by more than twice between the two groups. Such a fold-change threshold is commonly employed as a screening criterion for identifying differentially expressed substances in omics studies.

A heatmap of the Z-scores for lipid species across both groups was constructed to visualize expression patterns (Fig. 4D). Lipid species were categorized into three classes: glycerolipids (GL), glycerophospholipids (GP), and sphingolipids (SP). The results revealed distinct expression patterns between the two groups, with You450 samples showing higher levels of specific GL and SP species, while GP species were predominantly enriched in You150 samples. These patterns suggest differences in lipid composition and possibly underlying lipid metabolic activity between the two groups.

Among the 250 differentially expressed lipid species, the top 10 downregulated lipids were further highlighted based on the magnitude of their fold change values (Table 3). These lipids exhibited the most pronounced reduction in the You450 group compared to the You150 group, with log₂ fold change values ranging from –1.67 to –3.40. Notably, the majority of these top lipids belonged to the triacylglycerol (TG) class, including polyunsaturated species such as TG(18:2_20:4_20:5) and TG(16:0_22:6_22:6). In addition, two phospholipids—PE(18:0_22:5) and PC(20:4_16:1)—were also among the most significantly downregulated lipids. These findings indicate that both neutral lipids and membrane phospholipids were markedly reduced with age, suggesting alterations in lipid storage and membrane composition in older birds.

**Table 3:** Top 10 differential lipids in abdominal fat between 150-day-old and 450-day-old Beijing You chickens (ranked by absolute fold change).

Index	Compounds	Class I	Class II	VIP	P-value	Log2FC
LIPID-P-1234	TG(18:2_20:4_20:5)	GL	TG	1.60	1.63E-02	−3.40
LIPID-N-0346	PE(18:2_14:0)	GP	PE	1.42	1.17E-02	−2.44
LIPID-P-2347	TG(17:1_18:2_22:6)	GL	TG	1.22	1.53E-02	−2.00
LIPID-P-1240	TG(16:0_22:6_22:6)	GL	TG	1.81	1.00E-03	−1.89
LIPID-P-1235	TG(16:0_22:5_22:6)	GL	TG	1.88	1.41E-03	−1.84
LIPID-N-0285	PC(20:4_16:1)	GP	PC	1.39	4.07E-02	−1.81
LIPID-P-0783	TG(12:0_14:0_18:0)	GL	TG	1.99	1.77E-03	−1.75
LIPID-P-0778	TG(12:0_12:0_18:0)	GL	TG	2.08	8.55E-04	−1.72
LIPID-N-0403	PE(18:0_22:5)	GP	PE	1.78	4.42E-05	−1.71
LIPID-P-1218	TG(18:2_20:3_20:4)	GL	TG	1.94	4.68E-03	−1.67

Enrichment analysis showed that the 250 differentially expressed lipids between the You450 and You150 groups were significantly enriched in metabolic pathways, linoleic acid metabolism, arachidonic acid metabolism and alpha-Linolenic acid metabolism pathways (Fig. 4E and Supplementary Table 9).

### Differential analysis of abdominal fat lipids between the Ros150 and You150 groups

**Fig. 5:**
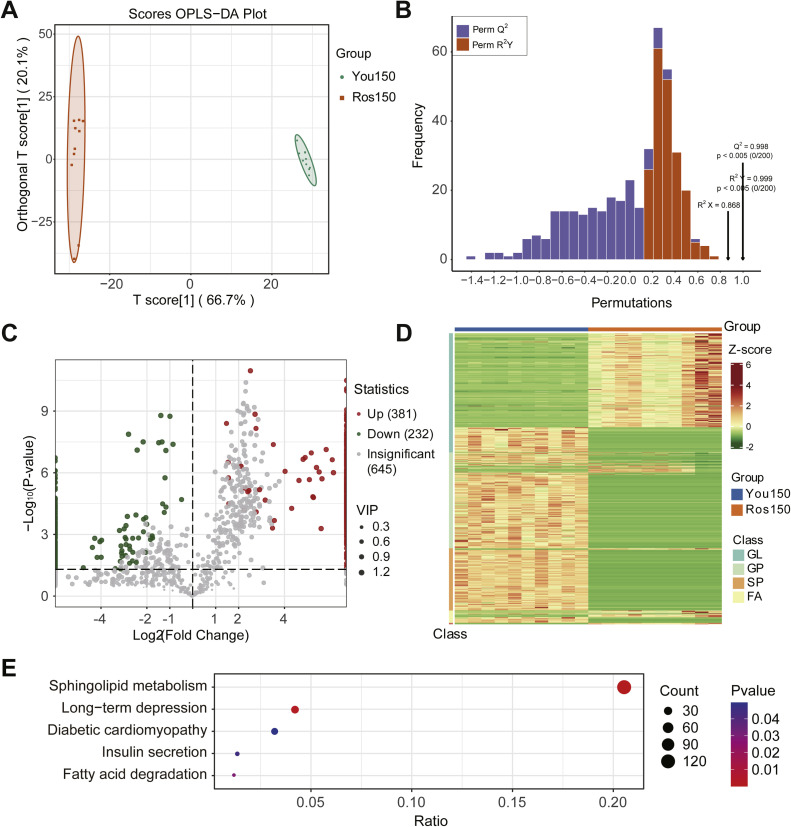
Differential lipids identification between Bejing You chicken at 150 days of age (You150) and Ross 308 broilers at 150 days of age (Ros150). A. Orthogonal partial least squares discriminant analysis (OPLS-DA): OPLS-DA model. B. OPLS-DA: the permutation test. C. Volcano plot of differential lipids between You150 and Ros150 group. D. Heat map depicting differential lipids in You150 vs Ros150. E. KEGG enrichment of the differential lipids detected between Ros150 group and You150 group. GL: glycerolipids; GP: glycerophospholipids; SP: sphingolipids; FA: fatty acids.

OPLS-DA was used to evaluate differences in lipidomic profiles of abdominal fat between Beijing You chickens (You150) and Ross 308 broilers (Ros150). The score plot (Fig. 5A) showed a clear separation between the two groups along the primary component T score[1] (66.7 %) and the orthogonal component Orthogonal T score[1] (20.1 %), indicating distinct differences in lipid compositions between the two groups.

Model validation using permutation tests confirmed the reliability of the OPLS-DA model (Fig. 5B), with R²*X* = 0.868, R²*Y* = 0.999 (*p* < 0.005), and Q² = 0.998 (*p* < 0.005). Differential analysis identified 613 significantly different lipid molecules between the two groups, with 381 lipids upregulated in Ross 308 broilers and 232 lipids upregulated in Beijing You chickens (VIP > 1, *p* < 0.05) (Fig. 5C and Supplementary Table 10). The 613 significantly different lipid molecules included 178 Ross 308 broiler-specific lipids, 347 Beijing You chicken-specific lipids and 88 lipid compounds shared by the two breeds. Among these 613 differentially expressed lipids, 610 lipids (including 381 upregulated and 229 downregulated lipids in the You150 group) exhibited more than a two-fold difference between the two groups. Of these, 178 lipids were specifically expressed in the Ros150 group, while 347 lipids were specifically expressed in the You150 group.

A heatmap analysis (Fig. 5D) was conducted to visualize the expression patterns of lipid molecules in the two groups. Lipids were classified into four major categories: glycerolipids (GL), glycerophospholipids (GP), sphingolipids (SP), and fatty acids (FA). Differentially expressed lipids were predominantly enriched in the GP and SP classes, indicating that these two lipid types contributed most to the observed group differences. Specifically, Ross 150 broilers exhibited higher levels of GP species, whereas Beijing You chickens showed increased levels of GL and SP species, reflecting distinct lipid expression profiles between the two groups.

The 613 differentially expressed lipids between Ros150 and You150 were significantly enriched in sphingolipid metabolism, long-term depression, diabetic cardiomyopathy, insulin secretion, and fatty acid degradation pathways (Fig. 5E and Supplementary Table 11).

### Shared and unique lipid differences across comparisons

**Fig. 6:**
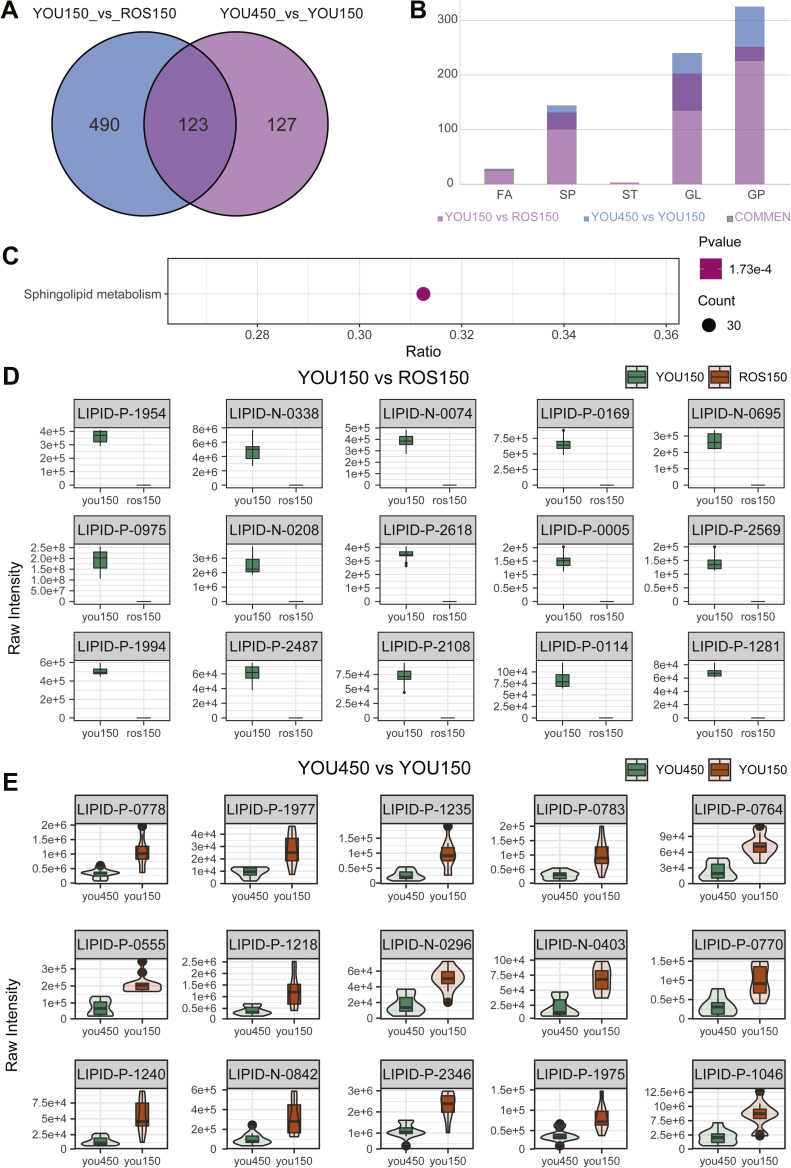
Comparative analysis of lipidomic profiles between experimental groups. A. Venn diagram illustrating the intersection of differentially expressed lipids between the You150 vs ROS150 and YOU450 vs You150 groups. B. Distribution and intersection of specific lipid classes, including fatty acids (FA), sphingolipids (SP), sterols (ST), glycerolipids (GL), and glycerophospholipids (GP), across the two experimental groups. C. Enrichment analysis of the intersecting differentially expressed lipids, highlighting significant pathways such as sphingolipid metabolism. D. Bar graphs depicting the actual concentrations of top significantly altered lipids in the You150 and ROS150 groups. E. Comparative bar graphs showing the concentrations of selected lipids in the YOU450 and You150 groups. Data are presented as mean ± SEM. Statistical significance was determined using appropriate tests (t-test, *p* < 0.05).

A total of 613 lipids were significantly different in the comparison of You150 vs. Ros150, while 250 lipids were differentially expressed between You450 vs. You150. Notably, 123 lipids were shared between the two comparisons, representing common differences across these conditions (Fig. 6A). The identified differential lipids were categorized into five major classes: fatty acids (FA), sphingolipids (SP), sterols (ST), glycerolipids (GL), and glycerophospholipids (GP). Among these, GP and GL exhibited the most prominent differences in both pairwise comparisons. Lipids common across the two comparisons were predominantly enriched in GP and GL classes, indicating their involvement in metabolic remodeling or functional differentiation across groups. (Fig. 6B). The 123 shared lipids were significantly enriched in the sphingolipid metabolism pathway (*p* = 1.73e-4) (Fig. 6C and Supplementary Table 12). Quantitative analysis of top differentially expressed lipids in the You150 and ROS150 groups (Fig. 6D) reveals substantial variations in their concentrations. Furthermore, comparative analysis of lipid concentrations between the You450 and You150 groups (Fig. 6E) provides additional insights into the impact of experimental conditions on lipid profiles. These findings collectively emphasize key alterations in lipid metabolism and identify potential biomarkers and pathways for further investigation.

## Discussion

This study provides a comprehensive comparison of slaughter traits, meat quality, tissue morphology, and lipid composition between Beijing You chickens and Ross 308 broilers, revealing significant differences in growth characteristics, fat deposition patterns, and lipid metabolism between the two breeds. These findings align with prior research on breed-specific variations in muscle and fat development and contribute to a better understanding of the genetic and metabolic mechanisms that underpin these differences.

### Comparison of slaughter traits between broilers with different ages and species

Our results confirm that Ross 308 broilers exhibit significantly higher body weight, carcass weight, and muscle mass compared to Beijing You chickens, which is consistent with commercial breeding goals aimed at maximizing muscle yield. Several studies have similarly reported that modern broiler breeds like Ross 308 broiler are selected for their rapid growth and high feed conversion efficiency, leading to increased muscle mass and decreased fat deposition (Tavárez and Solis, 2016; Kpomasse et al., 2021). These traits are essential for the poultry industry, where the focus is on producing lean meat for large-scale consumption.

Conversely, Beijing You chickens, known for their traditional and slow-growing nature, accumulate fat more efficiently, especially in the abdominal region. This observation mirrors previous study which noted that indigenous chicken breeds typically exhibit higher fat deposition compared to commercial broiler breeds (Kang et al., 2022). The differences in fat deposition observed in our study support the idea that Beijing You chickens may have evolved under selective pressures favoring fat storage, a trait that provides a buffer in less controlled, traditional farming environments. In comparison, broilers like Ross 308 broiler have been selectively bred for high yield, which prioritizes muscle growth over fat accumulation.

### Comparison of meat quality in broilers across different ages and species

The meat quality results further emphasize the differences between these two breeds. The breast and leg muscles of Beijing You chickens were significantly more tender than those of Ross 308 broilers, aligning with the findings of Ismail and Joo (2017), who reported that slower-growing, traditional breeds tend to produce more tender meat due to increased intramuscular fat and connective tissue breakdown. Additionally, the decrease in tenderness observed in older Beijing You chickens, likely associated with increased intramuscular fat deposition, is also consistent with the observations of the study, which observed that tenderness declines with age in poultry as fat accumulation increases. Our results corroborated these findings, showing that older Beijing You chickens exhibited higher shear force values, indicating reduced meat tenderness compared to younger birds.

In contrast, Ross 308 broilers exhibited less variation in tenderness across different age groups. These findings are in line with those of Wang et al. (2019), who discovered that commercial broilers, with their rapid growth and high muscle yield, tend to have consistently tender meat, which is one of the reasons they dominate the global poultry market. The relatively lower fat content in Ross 308 broilers likely contributes to their higher muscle tenderness, as fat tends to soften meat fibers and improve tenderness.

### Comparison of tissue morphology and fat deposition in broilers across different ages and species

The tissue morphology analysis revealed that Beijing You chickens, particularly the older birds, exhibited significantly larger abdominal fat cells compared to Ross 308 broilers, indicating a stronger capacity for lipid storage. This observation aligns with the previous studies, who reported that traditional breeds like indigenous chickens accumulate fat more efficiently in the abdominal region compared to broiler chickens (Ismail and Joo, 2017). The differences in fat cell size may be linked to genetic factors regulating lipid metabolism and storage. A study found that indigenous chickens accumulate more abdominal fat due to differences in adipocyte development and lipid synthesis pathways (Zmrhal et al., 2023).

The observed differences in muscle fiber diameter between Ross 308 broilers and Beijing You chickens reflect the distinct growth patterns and muscle development strategies between fast- and slow-growing chicken breeds. Ross 308 broilers, selected for rapid muscle accretion and high feed efficiency, exhibited significantly larger breast muscle fiber diameters compared to Beijing You chickens. This hypertrophic growth pattern is consistent with commercial breeding goals focused on maximizing muscle yield within a short production cycle. In contrast, Beijing You chickens showed smaller muscle fiber diameters, especially in breast meat, which is typically associated with finer texture, improved tenderness, and richer flavor—traits favored in traditional and niche markets. Notably, the breast muscle fiber diameter did not significantly increase with age in Beijing You chickens, suggesting that muscle growth may be driven more by fiber number (hyperplasia) than fiber size (hypertrophy), or that a growth plateau had been reached by 150 days. Interestingly, no significant differences were observed in leg muscle fiber diameters across the three groups, indicating that leg muscles may be less responsive to genetic selection and growth rate differences, or are influenced more by activity levels and load-bearing function than by breed or age. These histological differences provide a cellular basis for the contrasting meat quality traits between the two breeds and highlight the potential of muscle fiber characteristics as biomarkers for selecting lines with desirable meat quality traits in breeding programs.

### Insight into lipid composition and metabolism

In this study, we investigated the lipidomic composition of abdominal fat in Beijing You chickens at two key ages and compared it with that of the fast-growing Ross 308 broilers. The results demonstrated that breed-related differences in lipid composition far exceeded those caused by age.

Lipidomic analysis revealed significant differences in the abdominal fat lipid profiles between Beijing You chickens and Ross 308 broilers. Compared to Ross 308, which predominantly contained glycerophospholipids, Beijing You chickens exhibited a higher proportion of sphingolipids and glycerolipids. This finding aligns with previous studies (Zhang et al., 2020), which reported that indigenous chicken breeds differ from commercial broilers in lipid class composition, suggesting a tendency in traditional breeds to store fat primarily in the form of sphingolipids and glycerolipids, possibly associated with long-term fat storage.

The predominance of glycerophospholipids in Ross 308 broilers supports the notion that these lipids are primarily utilized for energy metabolism and membrane integrity rather than fat storage. Principal component analysis (PCA) and orthogonal partial least squares discriminant analysis (OPLS-DA) further confirmed the lipidomic differences between the two breeds, showing a clear separation between Beijing You and Ross 308 chickens. Similar results have been reported in other studies, highlighting distinct metabolic profiles among poultry breeds (Wang et al., 2017). Moreover, a total of 525 differential and breed-specific lipids were identified, offering potential candidates for future studies into the flavor differences between the two breeds or for meat product identification purposes.

Differences in fat deposition, muscle mass, and lipid metabolism between Beijing You chickens and Ross 308 broilers are of great relevance for poultry breeding and meat production. Our findings suggest that Beijing You chickens, with their stronger fat accumulation capacity, may help enhance meat tenderness and flavor—traits highly valued in premium markets. This aligns with previous reports (Yue et al., 2024), which emphasized the close relationship between meat quality traits (such as tenderness and flavor) and fat content, particularly intramuscular fat. In contrast, Ross 308 broilers, due to their rapid growth and high lean meat yield, remain the preferred choice for large-scale meat production.

A better understanding of lipid metabolic pathways in both breeds provides potential targets for improving meat quality through genetic selection and breeding strategies. Our lipidomic results offer valuable references for identifying genetic markers associated with fat deposition and muscle growth, facilitating more efficient, market-specific breeding programs. As suggested by Tan et al. (2022), regulating poultry lipid metabolism through genetic tools or nutritional strategies can improve both meat quality and production efficiency, especially in traditional breeds like Beijing You chicken.

Given the market preference among some consumers for older hens, this study also explored lipid composition differences in abdominal fat between 150-day-old and 450-day-old Beijing You chickens. Results showed that aging led to notable changes in lipid composition, with increased levels of certain glycerolipids and sphingolipids in older birds. This observation is consistent with findings by Li et al. (2024), indicating that lipid metabolic pathways become more active with maturation, particularly in fat storage. Additionally, with increasing age, 218 lipids were significantly downregulated and only 32 were upregulated. Among them, only 80 lipids showed more than twofold changes, mainly belonging to triglycerides and glycerophospholipids, with only a small number of sphingolipids involved. However, a large portion of lipids showing smaller fold changes (less than twofold) were sphingolipids, including various ceramide (Cer) subtypes—such as Cer-AP (11 types), Cer-AS (16), Cer-NS (4), Cer-NDS (1), Cer-NP (2)—and sphingomyelins (SM, 3 types) (see Supplementary Table 8). These sphingolipids are critical regulators of cell signaling, membrane stability, apoptosis, and metabolic diseases. Their significant variation suggests that aging adipose tissue may experience metabolic stress, inflammation, or cellular remodeling (Guerre et al., 2022; Nematbakhsh et al., 2021; Chen et al., 2023).

Further enrichment analysis revealed that age-related lipid differences were primarily enriched in the sphingolipid metabolism pathway, followed by fatty acid degradation and insulin resistance pathways. This suggests that aging in Beijing You chickens leads to significant changes in lipid synthesis, degradation, and signaling regulation in abdominal fat tissue. The enrichment of pathways associated with metabolic diseases, such as diabetic cardiomyopathy, also points to a possible imbalance in lipid homeostasis during later life stages.

A cross-comparison of breed- and age-related lipid differences identified 123 shared differential lipids. In addition to glycerolipids and glycerophospholipids, a substantial number were sphingolipids. These lipids were also significantly enriched in the sphingolipid metabolism pathway, further supporting its central role in fat deposition regulation. This part of the findings highlights the importance of lipid metabolic remodeling in the development and breed-specific traits of Beijing You chickens and offers a theoretical basis for further research on the molecular mechanisms and functional validation of key metabolic biomarkers involved in fat deposition.

## Conclusion and future perspectives

In summary, this study highlights the significant differences between Beijing You chickens and Ross 308 broilers in growth patterns, fat deposition, meat quality, and lipid metabolism. Beijing You chickens, known for their unique flavor and higher meat tenderness, primarily cater to niche markets that value taste, while Ross 308 broilers dominate industrial production due to their rapid growth and feed conversion efficiency.

Through integrated lipidomic and histological analyses, this research deepens our understanding of the metabolic pathways underlying fat storage and muscle development in poultry. Future efforts to elucidate the genetic basis of these phenotypic traits are expected to support molecular breeding strategies aimed at improving meat quality, fat deposition efficiency, and overall poultry production performance.

However, this study is limited by its lipidomics-only approach to understanding growth and breed differences in Beijing You chickens. Additionally, although the two breeds were raised under the same conditions for one week, their earlier growth environments and diets varied significantly, potentially influencing lipid metabolism and affecting the interpretation of results.

Future studies should incorporate more standardized feeding experiments and integrate multi-omics approaches—including transcriptomics, metabolomics, and genomics—to systematically uncover the molecular regulatory mechanisms of fat deposition in Beijing You chickens, thereby laying a more solid foundation for high-quality and precision breeding strategies.

## Disclosures

All authors disclosed no relevant relationships.

## Appendix Supplementary materials

Image, application 1

## Footnotes

Supplementary material associated with this article can be found, in the online version, at doi:10.1016/j.psj.2025.105613.
